# COVID-19: Current Status and Future Prospects

**DOI:** 10.3390/tropicalmed8020094

**Published:** 2023-01-31

**Authors:** Peter A. Leggat, John Frean, Lucille Blumberg

**Affiliations:** 1 World Health Organization Collaborating Centre for Vector-Borne and Neglected Tropical Diseases, College of Public Health, Medical and Veterinary Sciences, James Cook University, Townsville, QLD 4811, Australia; 2 School of Medicine, College of Medicine, Nursing and Health Sciences, University of Galway, H91 TK33 Galway, Ireland; 3 School of Public Health, Faculty of Health Sciences, University of the Witwatersrand, Johannesburg 2000, South Africa; 4 Centre for Emerging Zoonotic and Parasitic Diseases, National Institute for Communicable Diseases, Johannesburg 2131, South Africa; 5 Right to Care South Africa, Faculty of Veterinary Science University of Pretoria, Pretoria 0002, South Africa

This second Special Issue in a series of Special Issues in *Tropical Medicine and Infectious Disease* looks at recent global research on the current Coronavirus (COVID-19) Pandemic. The disease is caused by a novel virus: severe acute respiratory syndrome coronavirus 2 (SARS-CoV-2) [1,2]. The International Committee on Taxonomy of Viruses (ICTV) named the virus SARS-CoV-2, as it is genetically related to the coronavirus responsible for the SARS outbreak of 2003 [2]. While related, the two viruses are quite different in their behaviour. At the time of submission for publication (9 January 2023), COVID-19, named by the World Health Organization (WHO) on 11 February 2020, caused more than 657 million cases and over 6.6 million deaths with over 430,000 new cases within the past 24 h [2]. 

The COVID-19 pandemic greatly affected the capacity of health systems in providing essential health care [1], but in response, there has been a remarkable and timely development of vaccines and laboratory tests, including rapid antigen tests. There has been a rigorous application and promotion of public health measures in many countries around the world. As of 6 January 2023, there have been more than 13 billion vaccine doses of COVID-19 vaccines administered [2], although there remains a question concerning how equitable their distribution is. Our knowledge has expanded on the pathogenesis and treatment of COVID-19 and experience gained within different countries, and this is reflected in the enormous number of scientific papers generated, including those in this Special Issue. There have been 24 papers published upon peer review acceptance in this Special Issue, including 17 research papers [3,4,5,6,7,8,9,10,11,12,13,14,15,16,17,18,19], 2 review papers [20,21], 1 opinion piece [22], 1 commentary [23], and 3 systematic reviews [24,25,26]. Each paper in this Special Issue contributes to our understanding of COVID-19. 

The contributions of these 17 research papers can be summarized as follows. The first of the research papers aimed to explore the risk perception and prevention practices of coronavirus disease 2019 (COVID-19) among people living in high- and low-population density areas in Dhaka, Bangladesh. Interestingly, findings showed that participants were not concerned about COVID-19 and believed that coronavirus would not have a devastating impact on Bangladeshis; thus, they were reluctant to follow prevention measures and undergo testing [3]. The second study investigated the clinical features of severity and mortality among COVID-19 patients in Luanda, Angola. Fever (46%), cough (47%), gastrointestinal symptoms (26.7%), and asthenia (26.7%) were the most common symptoms. About 64.4% of the patients presented coexistent disorders, including hypertension (42%), diabetes (17%), and chronic renal diseases (6%) [4]. The third study assessed the characteristics, practices, and associated factors of self-medication (SM) by the public during the COVID-19 pandemic in Sargodha, Pakistan. Consciousness and understanding about the possible adverse effects of SM must be established and validated at a continuous level; in addition, at the commercial level, collaboration from pharmacists in not selling products (especially prescription-only medicines) without a certified prescription must be developed and implemented [5]. The fourth of these constructed a compartmental model with a time-dependent transmission rate that incorporates two sources of infection. The model was applied to the COVID-19 spread data from a university environment, namely, the Institut Teknologi Bandung, Indonesia, during its early reopening stage, with a constant number of students. The results show a significant fit between the rendered model and the recorded cases of infections [6]. The fifth of these analyzed the COVID-19 contact tracing dataset from 15 July to 31 December 2021 using multiple logistic regression analyses, considering exposure details, demographics, and vaccination history. Having symptoms, unprotected exposure, lower education level, and receiving low-potency vaccines increased the risk of laboratory-confirmed COVID-19 following healthcare-related exposure events. [7]. In the sixth of these, the cases and deaths for the four waves of COVID-19 in 119 countries and regions (CRs) were collected. They compared the mortality across CRs where populations experience different economic and healthcare disparities. The clinical outcomes in developing countries became worse along with the expansion of the pandemic [8]. The purpose of the seventh of these was to compare four commercial RT-qPCR assays with respect to their ability to detect the SARS-CoV2 virus from nasopharyngeal swab samples referred to Laboratorio Carvajal IPS, SAS in Tunja, Boyacá, Colombia. GeneFinderTM COVID-19 Plus RealAmp (GF-TM) and Berlin-modified protocols offer the best sensitivity and specificity, with similar results in comparison to the gold standard Berlin protocol [9]. The eighth of these explored the epidemiology of emerging variants of SARS-CoV-2 that circulated in Bangladesh from December 2020 to September 2021, representing the second and third waves. A rapid growth in the number of variants identified across Bangladesh showed virus adaptation and a lack of strict quarantine, prompting periodic genomic surveillance to foresee the spread of new variants, if any, and to take preventive measures as soon as possible [10]. The ninth was a document review of the health operations and technical expertise (HOTE) pillar coordination meetings’ minutes, reports, policy, and strategy documents of the activities and outcomes and feedback on updates on the HOTE pillar given at regular intervals to the regional incident management support team of the World Health Organization regional office for Africa. The coordination mechanism appeared to be robust; some challenges included the duplication of coordination efforts, communication, documentation, and information management [11]. The tenth of these was the use of a triage strategy of routine COVID-19 testing for febrile patients with viral prodromes. All febrile patients with viral prodromes and no epidemiologic risk for COVID-19 were first admitted to a designated ward for COVID-19 testing. During successive COVID-19 pandemic waves in a dengue-endemic country, coinfection with dengue and COVID-19 was uncommon. [12]. The eleventh of these was a descriptive longitudinal study conducted for determining the community transmission of SARS-CoV-2 in high- and low-density areas in Dhaka city. No differences in the seropositivity rates depending on the population gradient were observed [13]. The twelfth study was conducted to determine the effectiveness of the combined use of remdesivir and regdanvimab in patients with severe COVID-19. In patients with severe COVID-19, clinical outcomes can be improved by administering regdanvimab in addition to remdesivir [14]. In the thirteenth study, the authors compared excess all-cause mortality and COVID-19 mortality in 25 Peruvian regions to determine whether most excess deaths in 2020 were attributable to COVID-19. Most excess deaths in Peru are related to COVID-19 [15]. The fourteenth study aimed to assess the magnitude of and factors associated with depression and anxiety among Vietnamese frontline hospital healthcare workers in the fourth wave of COVID-19. There was a relatively high prevalence among Vietnamese hospital healthcare workers exhibiting symptoms of depression and anxiety during the ongoing pandemic [16]. The fifteenth study explored the association between body mass index (BMI), the prevalence of overweight and obesity, and the COVID-19 mortality rates in 25 Peruvian regions, adjusted for confounding factors, using multiple linear regression. As obesity prevalence increases, COVID-19 mortality rates increase in the Peruvian population ≥ 15 years [17]. The sixteenth study reported on an autochthonous outbreak of SARS-CoV-2 P.1 variant infections in southern Italy in seven subjects who had not travelled to endemic areas or outside the Apulia region. The circulation of variants of concern highlights the importance of strictly monitoring the spread of SARS-CoV-2 variants using genomic surveillance and by investigating local outbreaks [18]. The goal of the last study was to determine the frequency of newly diagnosed diabetes mellitus (DM) and its different types among COVID-19 patients and to check the glycemic control in diabetic cases for three months. COVID-19 patients with newly diagnosed diabetes had a high risk of mortality [19]. 

There were two review papers in this Special Issue. The first of these was a review examining the coagulopathy of dengue and COVID-19, particularly looking at clinical considerations [20]. The objective of the second review was to describe the intimate relationship between the gastrointestinal tract, including the liver and pancreas, and the pathogenesis, clinical course, and outcomes of the COVID-19 pandemic. Patients with gastrointestinal autoimmune diseases require close follow-up visits and may need modifications in immunosuppression. Acute pancreatitis is a rare manifestation of COVID-19, but it must be considered in patients with abdominal pain. [21]. There are two other papers in this Special Issue. The first is an opinion piece examining the possible consequences of the overlapping of pulmonary fibrosis secondary to COVID-19 and tuberculosis in the setting of sub-Saharan Africa, the region of the world with the highest prevalence of helminth infection [22]. The second was a commentary on COVID-19 vaccine hesitancy in sub-Saharan Africa. The authors’ overarching opinions were that political influences, religious beliefs, and low perceived risk exist in sub-Saharan Africa, and they collectively contribute to COVID-19 vaccine hesitancy [23]. There are also three systematic reviews. The first of these sought to assess breakthrough SARS-CoV-2 infections in vaccinated individuals by variant distribution and to identify common risk associations. It was found that continued mitigation approaches (e.g., wearing masks and social distancing) are warranted even in fully vaccinated individuals to prevent transmission [24]. The second systematic review aimed to assess the prevalence of people living with HIV (PLWH) among COVID-19 cases and whether HIV infection affects the risk of severe COVID-19 or related death at the global and continental level. Although there is a low prevalence of PLWH among COVID-19 cases, HIV infection may increase the severity of COVID-19 in Africa and increase the risk of death globally [25]. The last systematic review examined the risk of breakthrough infections in vaccinated individuals at a high risk of exposure, such as healthcare personnel (HCP). The authors’ findings further support the published high effectiveness rates of mRNA vaccines in preventing SARS-CoV-2 infections among fully vaccinated HCP [26].

The diversity of papers, the depth of the topics, and the relative geographical reach of the authors in this Special Issue confirm the continued collective major interest in COVID-19. There are 253 contributors for the 24 papers published in this Special Issue with affiliations in Europe, Africa, North America, South America, and Asia-Pacific. This wide-ranging open access collection contributes to a much better understanding of the epidemiology, presentation, diagnosis, treatment, prevention, and control of COVID-19. As the editors of this Special Issue, we trust that you find the content valuable, as the authors are pleased to share their knowledge with an international audience.

We currently have another opportunity to update advances in this field via a third Special Issue, “COVID-19: Current Situation and Future Trends”. We encourage you to publish your work in and/or propose a Special Issue for *Tropical Medicine and Infectious Disease*.
